# Reinforcement learned adversarial agent (ReLAA) for active fault detection and prediction in space habitats

**DOI:** 10.1038/s41526-023-00252-9

**Published:** 2023-02-13

**Authors:** Matthew Overlin, Steven Iannucci, Bradly Wilkins, Alexander McBain, Jason Provancher

**Affiliations:** Department of Autonomy and Data Science, PacMar Technologies, Honolulu, HI USA

**Keywords:** Mechanical engineering, Statistics, Applied mathematics

## Abstract

With growing interest for human space tourism in the twenty-first century, much attention has been directed to the robust engineering of Environmental Control and Life Support Systems in space habitats. The stable, reliable operation of such a habitat is partly achieved with an ability to recognize and predict faults. For these two purposes, a reinforcement learning adversarial agent (ReLAA) is utilized in this work. A ReLAA is trained with experimental data to actively recognize and predict faults. These capabilities are achieved by proposing actions that activate known faults in a system. Instead of issuing these harmful actions to the actual hardware, a digital twin of the mock space habitat is simulated to discover vulnerabilities that would lead to faulted operation. The methods developed in this work will allow for the discovery of damaging latent behavior, and the reduction of false positive and negative fault identification.

## Introduction

Space tourism is a budding industry with increased interest from the general public^1,2^. Companies such as SpaceX, Blue Origin, Virgin Galactic, and Boeing are either planning sub-orbital leisure flights or have already completed such trips. Union Bank of Switzerland estimates that the space tourism sector of the space economy will be worth US$4bn by 2030^3^. Present-day trips, however, may only be short-duration visits that occur over a period of hours or days. For such trips to be possible, science and engineering research has sought to understand the potential for medical risk during these crewed missions^4^. To increase the safety and reliability of these missions, accurate system health monitoring (SHM) must be deployed. The methods in this work will identify faulted operation in order to enable safer leisure travel with reduced medical risk.

Separate from the design, engineering, and construction of vessels launched into low Earth orbit, this work primarily seeks to monitor the operation of these vessels. Many conventional SHM fault detection methods compare measurement data with established healthy operational bounds^5–9^. Such methods may be described as passive, since a fault is declared based on static pre-defined rules. Passive HM does not capture and understand the short- or long-term dynamics of the system, thus leaving it vulnerable to unexpected or sudden faults. For example, a passive system will not understand the relationship between two features that could combine into a coupled or cascading fault.

Other research has developed fault detection solutions that can be updated during operation, but these methods may be purely data-driven models^10–12^. Because the agent in this work employs a physics-based digital twin, potential failures may be captured in a digital twin’s simulation results. Such information is useful during the agent’s training and deployment phases. Purely data-driven models may not have failure data available during training. Such failure data may not be easily attainable from an experimental setup with expensive hardware assets^8^.

Prior research has found that purely physics-based models or hybrid physics-based and data-driven models hold certain key advantages not found with purely data-driven modeling approaches^13,14^. In short, models with physical basis are understood to be more explainable, generalizable, and interpretable; all are qualities necessary in models of life-sustainment systems. Other digital twin systems have been successful in integrating physics-based models in lieu of a surplus of data that is necessary for supervised machine-learned systems^7,15^.

Each ReLAA developed and implemented in this work is an artificial neural network (ANN). Such networks are often trained with variants of gradient descent (ADAM, SGD, etc.), a first-order optimization algorithm used to find local minima in objective functions. Instead of these traditional optimization algorithms, some have found advantages with training ANNs through neuroevolution, an evolutionary process that allows an ANN’s parameters to change with new training data. Unlike gradient-based approaches, activation functions, hyperparameters, architectures, and algorithms can be learned in addition to the ANN parameters^16,17^. As explained later in this article, the training process for the ReLAA is completed through neuroevolution.

Artificial intelligence is usually implemented in the form of an ANN which may be described as a universal approximator. They are trained through a learning process where the parameters of the ANN are optimized to approximate a unique policy. Through reinforcement learning, a perturb-and-observe approach is used during the training process^18^. Actions selected by an agent are issued to a digital twin of an experimental apparatus (or an actual experimental apparatus), and the results are quantified as desirable or not by calculating a reward. If the actions lead to a large reward, then the ANN yielding the high reward is used to generate offspring agents^19^. In this work, a ReLAA is not used for controlling elements in the mock space habitat, but instead for fault recognition and prediction.

Unfortunately, genetic algorithms like NeuroEvolution tend to converge upon a single solution^19,20^. In a system as complex as the one designed and implemented in this work, a single solution is not capable of understanding the varied dynamics while still being computationally feasible^21^. Research into expanding the diversity of the solution space is ongoing, with promising results^17,20,22^. For the ReLAAs, two common techniques are implemented, the clustering and fitness sharing, both of which are designed to promote diversity and outliers during training.

This work introduces a framework to develop and test an active fault detection strategy on a physical demonstration system. First, in Methods section, the design and construction of the physical mock space habitat for life sustainment is outlined as well as the sensors and tools required to measure and communicate the physical system state to the software implementation. Also, in Methods section, a brief description is given for the following project tasks: middleware implementation, digital twin development, fault elicitation and reinforcement learning. In Results section, results are shared from the experimental operation and fault emulation. Results from the deployment of multiple ReLAAs are also presented. Finally, a discussion and conclusion are included.

## Methods

### Physical demonstration system

Each ReLAA developed in this work was tested and validated with live sensor stream data collected from the mock space habitat. The experimental setup is described as a system of systems: thermal control system (TCS), grey water filtering system (GWS), and an electrical integration model.

The goal of the physical demonstration system is to have a physical test system capable of eliciting measurable, realistic faults that are representative of an actual space habitat. Considerations in the design must be made for not only the actuation of faults within the system(s) but also the measurement and detection of the faults. The measurement and detection capability, primarily achieved with a variety of sensors, allows for the physical system to be integrated with a physics-based model to create a unified digital twin. The large volumes of data, collected via instrumentation hardware in the mock space habitat, will be key in training the ReLAAs and also validating the digital twin used by the ReLAAs.

An empty, isolated room was re-purposed to serve as the mock space habitat in this work with an assumed volume of 28 m^3^ and 9.3 m^3^ of habitable space per occupant. Some prior work has investigated many of the factors that would lead to a certain habitable volume, and has suggested a lower limit for the habitable volume given a certain number of days for a crewed voyage^23^. A volume of 9.3 m^3^ would roughly translate to a crewed duration of 17 days (or fewer). Thus, the decision was made to consider a habitat capable of sustaining three personnel. Then, the number of habitat occupants (3) was used as the basis for sizing the GWS. Waste produced by each occupant is assumed to be 7L/day/person. With the assumption that the GWS would be processing a day’s volume of water in 1 h, a through-flow rate of 21 L/h is assumed. The estimated maximum power consumed by the whole GWS is 250 W. The room’s temperature would be controlled with the TCS so that a habitat temperature of 20 °C is maintained. Altogether, a maximum power of 560 W is assumed from the TCS. These design decisions spurred an initially estimated power draw for each system to ensure appropriate relative power draws and the appropriate consideration of components. These power draws were then used to size the electrical system. Given 250 W from the GWS, 560 W from the TCS and 200 W from a load bank, the electrical system was sized to supply 1 kW of power to the whole mock space habitat.

As the scope of this project is limited to the aforementioned systems, other potential systems typically found in an Environmental Control and Life Support Systems (ECLSS) will be emulated in the experimental setup with the 200 W load bank. The load bank was sized to account for the difference between the GWS and TCS power draws and the capacity of the DC power source to ensure that all habitat systems cannot be powered simultaneously. This will enable faults to cascade when the system is placed in states where power consumption is approximately equal to the system’s capacity.

### Thermal control system (TCS)

The TCS was designed, built, instrumented, and operated as one of the sub-systems in the mock space habitat. The TCS in this work is different from more practical systems which may be integrated with ECLSSs in modern spacecraft^24^. Certain assumptions were made and should be noted. There is a single closed loop of circulating water, which would not be practically implemented on spacecraft. Typically, there would be internal and external loops, and other fluids would be circulated through these loops such as a propylene glycol mixture or ammonia which have lower freezing temperatures. Because this TCS is to be used as part of a mock space habitat for the ReLAA, simplifications in the TCS design were accepted. With the current design and implementation, a variety of faults could still be realized in the experimental setup.

**Fig. 1:**
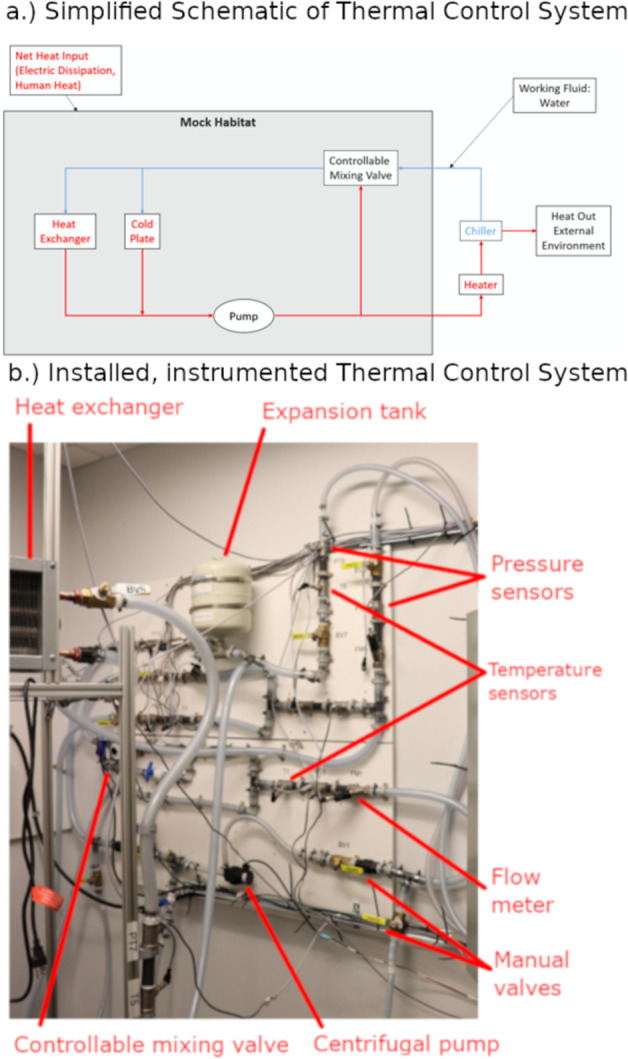
In the thermal control system (TCS), water is circulated through a closed loop to regulate the mock space habitat’s room temperature. **a** A simplified schematic illustrates the operation of the TCS by showing how important components (heat exchanger, pump, chiller, and heater) are arranged in the loop. **b** The TCS was mostly assembled, installed, and instrumented on one wall within the mock space habitat (chiller not shown). (The annotated picture is provided by PacMar Technologies and used with permission.).

In the TCS, the temperature measurements are insightful since the goal of the TCS is to regulate the habitat’s room temperature. The habitat temperature is ultimately regulated with the proper operation of the TCS, and this is achieved with additional observability from other sensors. Pressure and flow measurements obtained at various locations in the TCS allow for sufficient visibility. An annotated picture of the TCS is shown in Fig. 1.

### Grey water filtering system (GWS)

**Fig. 2:**
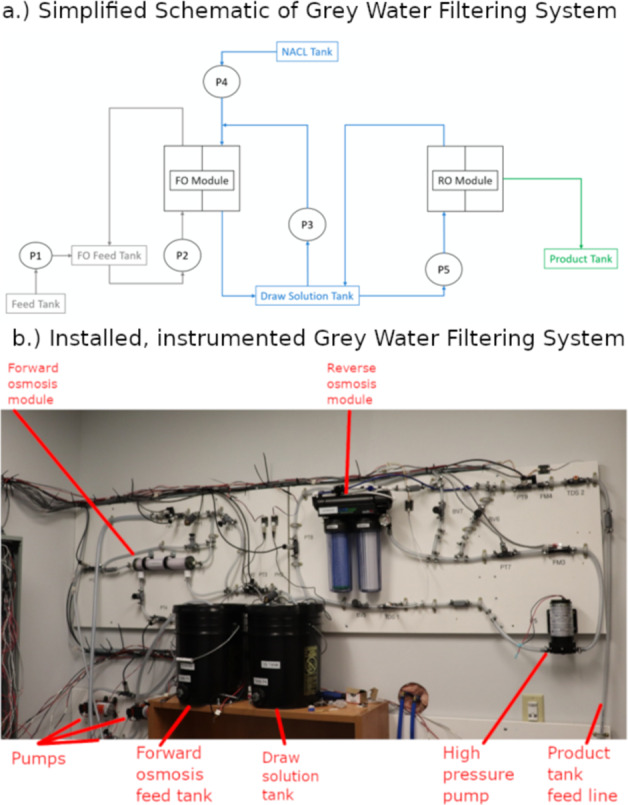
In the grey water filtering system (GWS), grey water is sourced from a feed tank, filtered through a forward osmosis (FO) module, filtered through a reverse osmosis (RO) module, and finally fed into the product tank as potable water. **a** A simplified schematic illustrates the operation of the GWS. Essentially, there are 3 loops in which water flows. The FO and RO modules are key components. **b** The GWS was mostly assembled, installed, and instrumented on one wall within the mock space habitat. The GWS’s feed tank (left) and product tank (right) are out of view in this picture. (The annotated picture is provided by PacMar Technologies and used with permission.).

The GWS was designed based on a water recycling system designed and operated in prior NASA work^25^. The components in the GWS were sized to deliver roughly 21 L/h of potable water. A simplified schematic of the GWS is shown in Fig. 2. When filtering grey water, the forward osmosis (FO) module is first used. Water flows through two different paths within the FO module, an inner path (consisting of feed/dirty water) and an outer path (consisting of draw/salt water) in the opposite direction. These two cavities containing water flow are separated by a semi-permeable membrane. Due to the osmotic pressure differential between the feed water and draw water, water passes through this membrane from the feed to draw side and ultimately into the draw solution (DS) tank. A majority of the contaminants would be removed in this filtering process with the resulting product from the FO being a salt water solution.

Water is then pumped out of the DS tank and through a reverse osmosis (RO) module. The RO module uses hydraulic pressure rather than osmotic pressure to force a solution through a semi-permeable membrane. The RO has one input and two outputs, with potable water exiting one output and rejecting water cycling back to the DS tank. For every 1 L of potable water flowing into the product tank, 2 L of water re-enter the DS tank from the rejection of the RO module. A variety of sensors are included in the experimental setup for the GWS: pressure, flow, total dissolved solids, electric power consumption, and tank water level.

### Experimental instrumentation, data acquisition, and control

The experimental setup integrates the data collection and operation activities of the physical demonstration system to a sensor and computational hub that can remotely monitor and actuate the system. This computational hub uses NASA’s core Flight System middleware software architecture to ingest sensor data, process raw voltages to physical values, and record historical data for use by a digital twin. Further developments were also accomplished to allow a terminal user to have remote access to the system, control programmable actuators on the system, and synchronize data to a cloud storage service. The sensor and control hub computer is a Raspberry Pi 4b single-board computer that communicates with two Arduino Mega microcontroller boards via USB. One microcontroller is used for reading sensor values, and the other is used for issuing commands to the demonstration system’s programmable actuators.

### Digital twin development

The integrated physics-based model (TCS, GWS, and electrical subsystem model), simulated in the MathWorks Simscape environment, is referred to as a digital twin because it is a virtual representation of the mock space habitat that is updated from real-time data and used to inform decision-making processes (fault recognition and prediction)^26,27^. In this work, the integrated model’s parameters were adjusted so that its simulation results would agree with experimentally captured data from the habitat. Because the ReLAA would need the ability to forward-look and anticipate faults, the digital twin would need to execute simulations faster than real time. The difference between simulated and measured waveforms was quantified, on average, with a mean absolute deviation of 7% or less. There is often a tradeoff between model accuracy and simulation speed, and this work prioritized simulation speed, though the model’s accuracy was found to be satisfactory.

Using the Functional Mock-up Interface (FMI) standard, the model was compiled as a functional mock-up unit (FMU) for use by a ReLAA^28^. The FMI standard is often used to simplify the creation, storage, exchange, and use of dynamic system models so that they may be flexibly simulated on a variety of computational platforms. When using the FMU, the ReLAA would specify input stimuli, set points, and other necessary information needed to specify a what-if scenario. With the integrated digital twin model implemented as an FMU, the ReLAA could be deployed offline to recognize faults in previously captured data or online to forward-look from a present snapshot of data.

### Fault elicitation

A failure modes and effects analysis was conducted to identify faults of interest within the system(s). These faults inform where and how perturbations are applied to the mock habitat and how they will be measured, and the expected system response given the operational scenario. Table 1 highlights some faults to be emulated in the mock habitat and the associated perturbation mechanism. For binary mechanisms like switches and relays, the agent has the ability to toggle the position between ON and OFF states. For continuously adjustable values such as variable resistors and values the agent had the ability to discretely increment the actuator by a set value. That is, the rightmost column in Table 1 lists the actuation points in the experimental setup where the ReLAA may affect change with its actions. The faults and mechanisms outlined in Table 1 were evaluated individually to uncover specific fault responses, as well as combinations of perturbations to elicit cascaded faults. This fault analysis informed a design of experiments that was later executed as part of this work.

**Table 1:** Identified faults through the failure modes and effects analysis and their associated mechanisms where the ReLAA can affect change in the experimental setup with its actions.

Subsystem	Faults	Mechanism
GWS	Membrane fouling, clogging, restrictions, leaks	Valves
TCS	Freezing	Chiller, external heater
	Blockages, leaks	Valves
Electrical	Power spikes	Variable resistors
	Shorts	Circuit breaker
	Power loss	DC power supply
Sensors	Sensor failure	Disconnect from power, software logic
	Sensor drift	Variable resistors, software logic
Misc. LSS systems	Load spikes	Load bank

### Reinforcement learning

This framework uses reinforcement-learned adversarial agents to learn perturbations to the digital twin that cause faults. The adversarial agent executes forward simulations while performing these perturbations on the digital twin to predict faults early, thereby identifying latent conditions which could lead to future faults. The adversarial agent is trained using a neuroevolutionary approach to learn how to cause and diagnose faults in the system. Here, a neural network represents the learned policy while an evolutionary algorithm iteratively optimizes the policy. The validated digital twin allows for the use of a genetic algorithm for training. Without it, an intractable amount of experimentally captured data from the mock space habitat would be necessary. The digital twin allows for the agents to break the system during training while not actually harming any physical systems. The verification of the demonstration system and the speed of simulation become essential to the quality of the adversarial agent.

In addition, fitness sharing is introduced after clustering to increase diversity in the population. In fitness sharing, each population member scales its fitness based on its proximity to population members. Therefore, densely packed population members have a lower fitness value than comparably good solutions in sparsely populated regions. The distance is the KL divergence between solutions, each represented by a discrete distribution based on states^29^. In general, the KL divergence between two probability distributions *P* and *Q* is computed as shown below in Eq. (1):

1 $${{{\rm{KL}}}}(P,Q)=\mathop{\sum}\limits_{i}p(i)\ln \left(\frac{p(i)}{q(i)}\right)$$

The policy calculates a discrete distribution based on the current state, therefore to compute the KL divergence between two policies, denoted by *π*_*P*_ and *π*_*Q*_. A set of recent states, *s* ∈ *S*, is used to get divergence by running the population member through an optimization problem. That is, the quantity computed in Eq. (2) is to be maximized to ensure diversity between two policies *π*_*P*_ and *π*_*Q*_.

2 $${{{\rm{KL}}}}({\pi }_{P},{\pi }_{Q})=\frac{1}{| | S| | }\mathop{\sum}\limits_{s\in S}{{{\rm{KL}}}}({\pi }_{P}(s),{\pi }_{Q}(s))$$

Next, a population member’s fitness scales with respect to the distance of all nearby population members. Therefore, with the KL divergence metric, the fitness function is scaled by *γ*, which is computed as shown in Eq. (3):

3 $$\gamma =\mathop{\sum}\limits_{Q}\{{{{\rm{KL}}}}({\pi }_{P},{\pi }_{Q})| {{{\rm{KL}}}}({\pi }_{P},{\pi }_{Q})\, <\, \delta \}$$

This scaling factor *γ* is applied such that the *i*th agent has a fitness of *F*_*i*_, but a scaled fitness of *F*_*i*_*γ*^−1^. This scaled fitness accounts for diversity between agents and is the metric used to select those agents which will parent subsequent generations of agents or be used in deployment. If a population member Q is within *δ* KL divergence it is considered a neighbor and included in the distance scalar. Population members that are similar to each other will not survive to the next generation, increasing diversity in the population.

The agent learns a mapping from the state space to the action space using guidance from a fitness function. The state space of the adversarial agent is the sensor data that would be mirrored in the real system. The action space is the set of components that the agent could perturb to cause a fault. For example, this includes each of the pipes it can clog and the filters it can foul. Each agent is a feed-forward neural network with inputs of the state-space sensor measurements, a list of 50 values. It has four layers, with a SoftMax output layer that has the same length as the number of possible faults, an array with 14 units. The output vector contains floats from 0 to 1 that represent the percentage probability that an action is taken. Then, when assuming a normal distribution, an action is selected. After this selection, the action is then issued to the digital twin or the mock space habitat. When the action is issued, a fault may or may not occur. If the ReLAA is well-trained (indicated with a high fitness), then it is more successful in causing faults with the actions selected from its output vector of probabilities.

Before training, a wide variety of experiments were performed to capture several possible healthy and faulted operational scenarios. In this data which shows actions and sensor data together, the ReLAA learns this mapping. That is, the agent was trained to find actions that push the system into failure. Agents only act during a limited time horizon to encourage the discovery of imminent failure cases.

The digital twin can be simulated with a real-time factor of 20 to 1. To train agents efficiently, each training run is conducted on a standalone CPU process, allowing for 24 branches to be simulated at once. Due to the total policy of the agents being spread over the entire population, parallelization allows for the current state space to be subject to numerous operational scenarios, leading to varied possible fault generation events. Overall, the parallelization enables training to be conducted up to 18× faster than without parallelization.

**Fig. 3:**
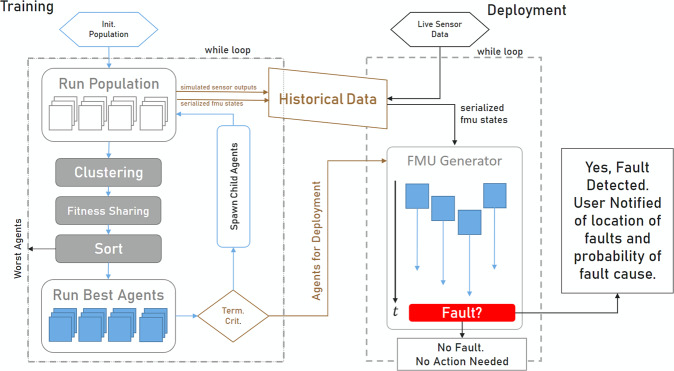
Multiple ReLAAs are trained before they are deployed to detect faults.

The flow of data during training and deployment is shown in Fig. 3. The iterative neuroevolutionary optimization occurs in the training block on the left, while data generated by agents is saved for future use in the deployment. Once the agents are trained, each can be loaded into the deployment framework where it will be given the chance to perturb simulation events from the current simulation state, as indicated in live sensor data provided to a deployed ReLAA. Note that the ReLAA can be provided with past or present sensor data and a digital twin simulation with 0 time steps can be performed to identify past or present faults. Of course, the results of the 0-time step simulation are trivial. From the digital twin’s initial state, the simulation would illustrate the time evolution into another state- the same state since the simulation is performed for 0 time steps. The simulated data is then analyzed to identify faults. A database of historical data is generated during the training phase due to the limitations of the FMU software standard and sensors. In order to generate a simulation from a complex state, the sensor values are fed into a Ball Tree algorithm. This structure finds the closest internal state that can be loaded into the FMU given the current sensor state. The FMU (the implementation form of the digital twin) is then simulated to identify faults and appropriately notify a user.

### ReLAA’s rewards and fitness

The fault detection methods shared in this work, using ReLAAs, will identify actions that lead to faulted operation. This is achieved utilizing a digital twin rather than taking potentially harmful actions in the mock space habitat. The ReLAA agent is adversarial in nature and earns a reward (during its training process) when damaging actions are found. The reward function at any given state, *r*_*s*_ is defined below where, *m*, is the number of features measured. For each feature, there is a reward. For a single feature *x*_*v*_, the feature reward depends upon if this feature value is above, below, or within upper and lower operational bounds. This is true for all features when the current state value is within the features lower bound and upper bounds, *x*_*l*_ and *x*_*u*_. The equation below is used to compute a feature reward.

4 $${r}_{v}=\left\{\begin{array}{ll}1,\quad &{{{\rm{if}}}}\,{x}_{v}\, >\, {x}_{u}\\ 1,\quad &{{{\rm{if}}}}\,{x}_{v}\, <\, {x}_{l}\\ \max \{\frac{{x}_{u}-{x}_{v}}{\frac{1}{2}({x}_{u}-{x}_{l})},\frac{{x}_{v}-{x}_{l}}{\frac{1}{2}({x}_{u}-{x}_{l})}\},\quad &{{{\rm{otherwise}}}}\end{array}\right.$$

Then, the reward function for a particular state *r*_*s*_ can be computed. *r*_*s*_ depends on all *r*_*v*_ values as shown in the equation below:

5 $${r}_{s}=\mathop{\sum }\limits_{v=1}^{m}\frac{1}{100}(1-{r}_{v})$$

Finally, the fitness of a given agent, *F*_*i*_, depends on all rewards *r*_*s*_ in a simulation run.

6 $${F}_{i}=\frac{1}{| | S| | }\mathop{\sum}\limits_{s\in S}{r}_{s}$$

The fitness for one ReLAA is ultimately the metric used to judge whether or not the ReLAA is used to be deployed or worthy of parenting subsequent generations in the neuroevolutionary process.

### Reporting summary

Further information on research design is available in the Nature Research Reporting Summary linked to this article.

## Results

The mock space habitat is operated in a variety of conditions to emulate the normal and faulted operations of an actual space habitat.

### Mock space habitat operation and fault emulation

**Fig. 4:**
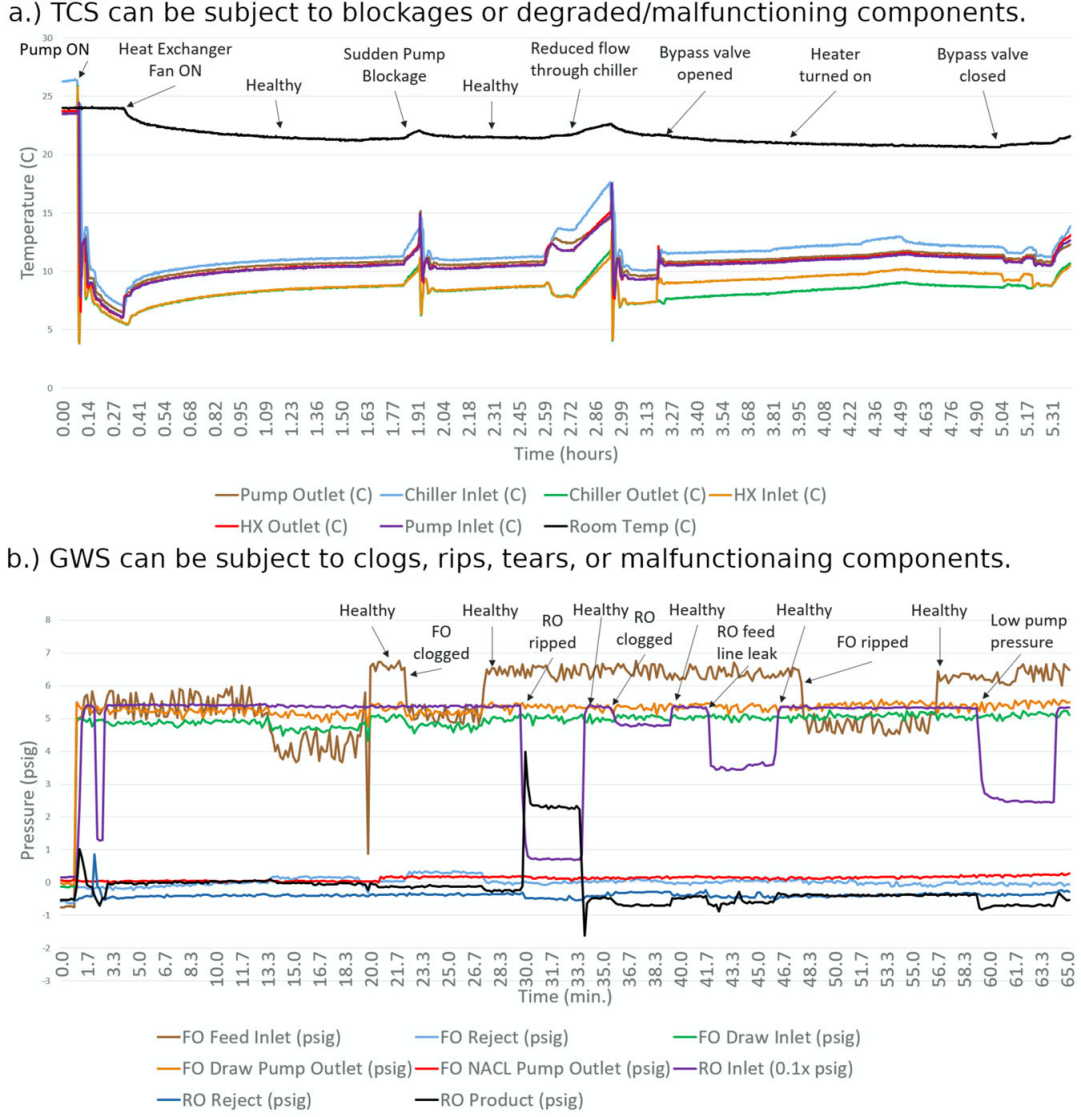
Experimentally captured data from the mock space habitat illustrate the operation during normal and faulted conditions. **a** Normal and faulted operation is shown for a variety of faults emulated in the TCS. **b** Pressures throughout the GWS change in response to normal and faulted conditions.

Twenty different experiments were conducted, each containing several disturbances to allow the mock habitat to operate in different states. Measurement data from the TCS and GWS are shown in Fig. 4.

First, an experiment was designed and performed over several hours with regard to the TCS. The time evolution of the temperatures in the TCS is shown in the first plot in Fig. 4. The TCS is allowed to reach a steady state, but then certain faults are emulated: sudden pump blockage, reduced flow through chiller, bypass valve opening/closing. When such faults are actuated, there is usually a deviation in the room temperature, which is undesirable.

Second, an experiment was performed with regard to the GWS, and a relevant plot of pressures is shown in Fig. 4. In the GWS, its normal or faulted operation can be visualized in these pressure measurements obtained at various points in the experimental setup. During healthy operation, the pressures are generally the largest. With the presence of a clog, leak, or other damage, several pressure measurements typically deviate from their nominal values. Measurement data were collected during various testing conditions—healthy, drifted, or faulted—allowing for an operation baseline to be established for agent training and deployment.

### Fault detection and prediction

Training was conducted on a validated digital twin, assessing 36 agents per epoch. On each generation, agents were sorted into a goal of six clusters, with each top 2 performing agents in each cluster chosen as parents for the successive generation. Each agent’s parameters were subject to Gaussian noise with a maximum variability of 0.25 with a standard deviation of 0.10 (each parameter is between –1 and 1). From 12 parents, a total of 36 agents were to be tested in the next training epoch. The performance of an agent is quantified as the cumulative sum of the rewards over the 10 testing runs of that generation.

**Fig. 5:**
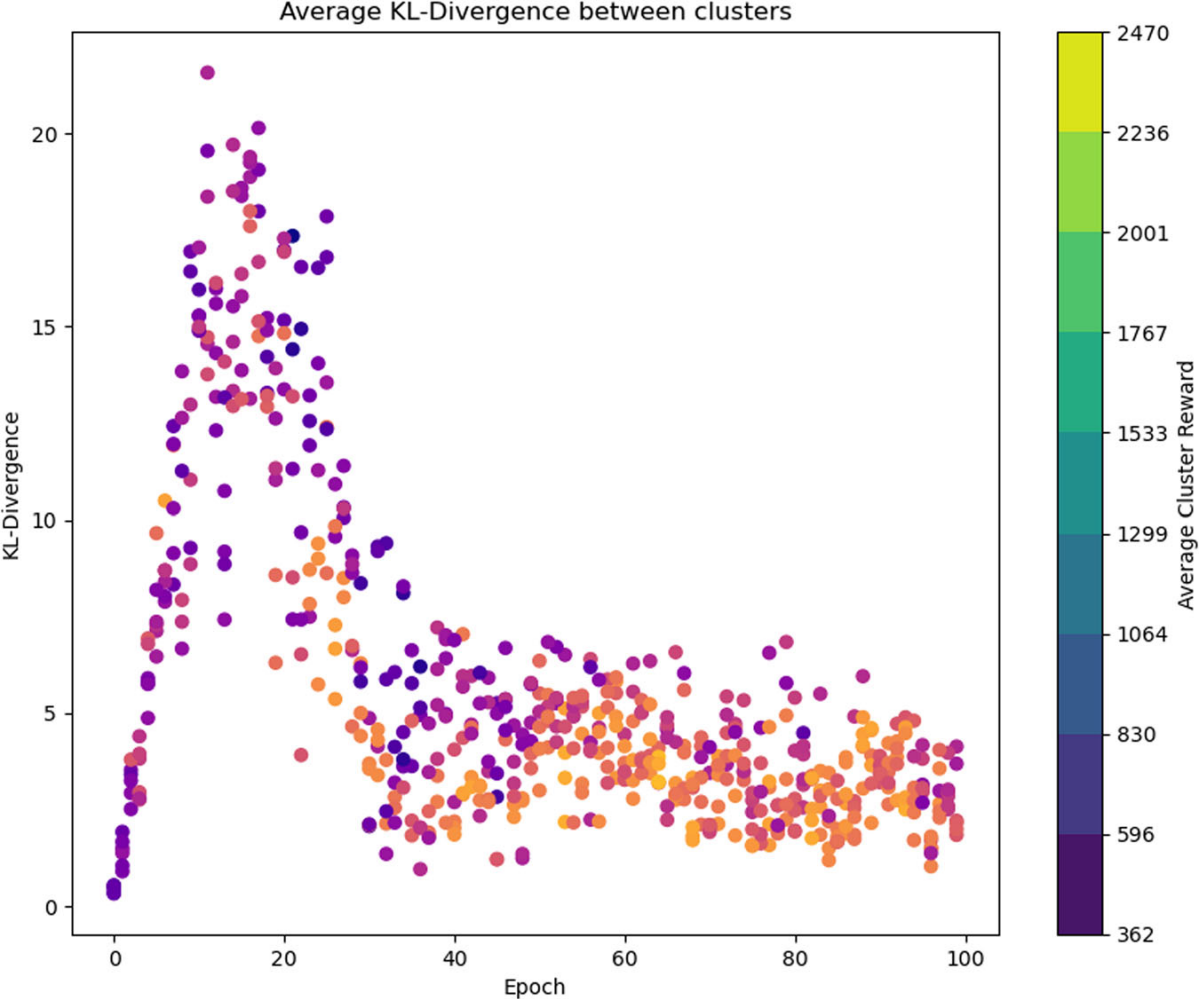
KL divergence during adversarial agent training. Average reward is represented by the dot color, with lighter being higher performers.

The fitness of the top performer in each generation is shown in Fig. 5. Over the scheduled 100 generations, the performance of the top agents steadily improved until the 65th epoch where the population reached its maximum reward. After this peak, the next 40 generations show a leveling-off in performance as agents find optimal solutions. In addition, the diversity of each agent is calculated at each generation. Some distance between clusters is desirable and incentivized through the neuroevolutionary structure. Relative distances between clusters increase by a factor of 20 throughout the first 20 generations as the system performs its state space exploration. With many agents trained to discover faulted behavior, the pool of agents used for fault detection is considered diverse. Following a peak in diversity around the 20th epoch, the clusters begin to approximately converge again to a steady state distance. During this time, the fitness of each cluster is improving as denoted by the lightening of each dot’s color.

**Fig. 6:**
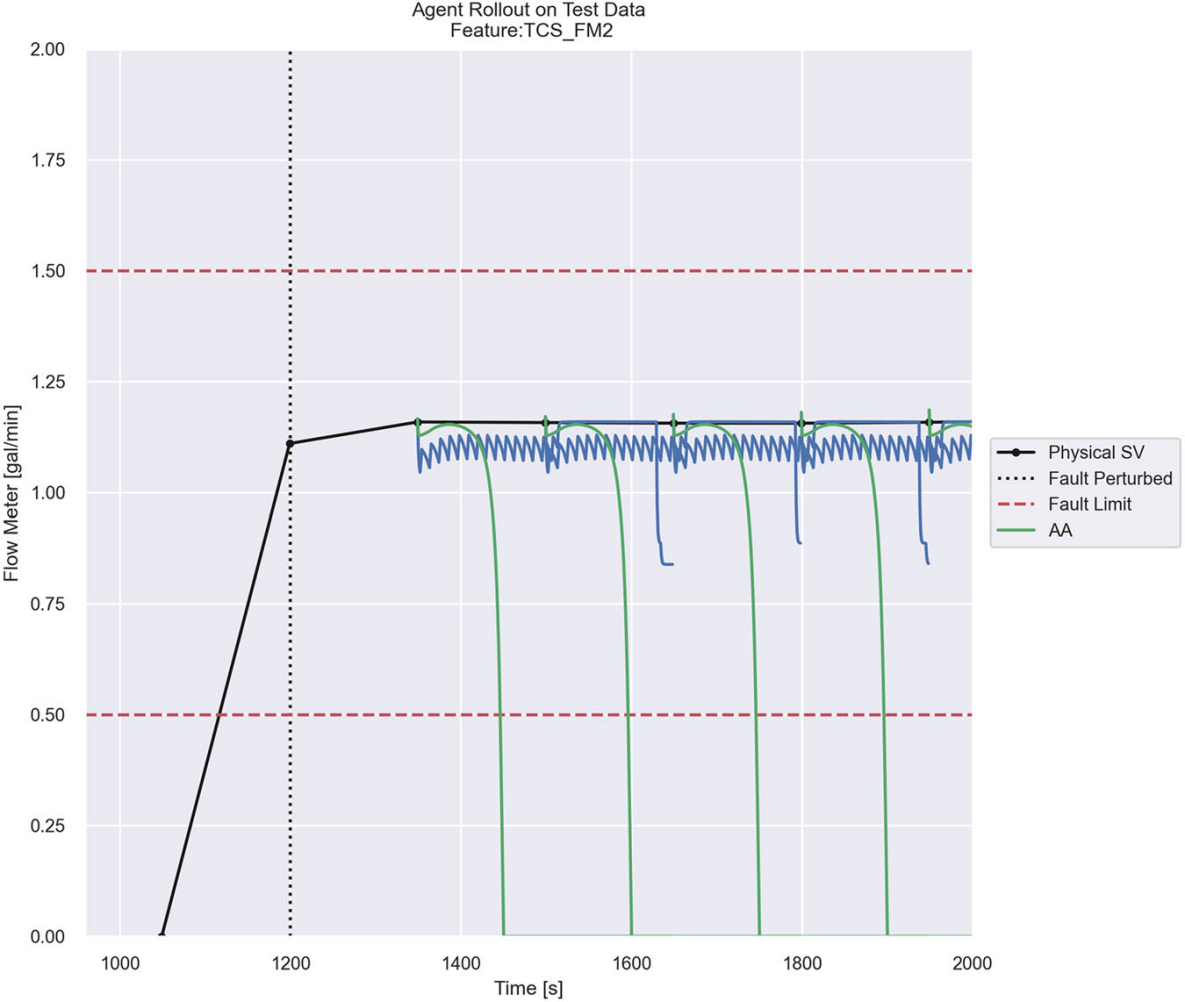
ReLAA rollout shown for TCS flow meter 2. A successfully trained ReLAA issues actions to the system which allow for faulted operation: the reduction in flow (outside of satisfactory operating bounds shown in red) throughout the TCS as shown in green.

When properly trained, the agents will issue actions to the system which leads to faulted behavior. For example, Fig. 6 shows an agent’s rollout in an operational scenario. As the flow meter 2 sensor measurement (shown in black in Fig. 6) would indicate, the TCS in the mock space habitat is operating satisfactorily, within healthy operating bounds (shown in red in Fig. 6).

When testing two of our agents on simulation-derived training data, both are able to force the system to a fault using two separate actuation methods. In the testing scenario, a clog within the RO module is actuated at a rate of 0.5% per second until it reaches an over-fault percentage of 30. This slight disturbance is recognized by the adversarial agents, and they each present a unique solution.

Agent 1 (green rollout in Fig. 6) actuates the RO clog fault within the GWS, causing a cascade effect through the electrical system. Because of the RO clog in the GWS, reduced flow is observed throughout the TCS. As the clog in the RO filter becomes worse, pressure grows above the control threshold level, triggering the pumps to activate. The GWS pumps draw more power than typical to push water through the clog. This results in a decrease in the habitat’s DC bus voltage. With a lower operating voltage for the TCS’s centrifugal pump, the pump cannot provide a sufficient pressure differential. For this reason, flow is reduced, and the room temperature cannot be effectively regulated with the TCS. In this particular example of an indirect fault, a clog in the GWS has indirectly prohibited the TCS from regulating the habitat temperature.

Agent 2 (blue rollout in Fig. 6) simulates the clogged strainer within the TCS. This filter is located on the intake of the TCS pump. As the clog builds, pressure slowly drops as there is a linear downtrend to the blue, jagged output. When the clogged strainer fault is further actuated, it is not the ideal choice to break the system. This demonstrates a key contribution to this active learning framework. Multiple underlying component issues, where none are independently causing a component fault, can lead to a full system fault. As demonstrated in this work, our active fault detection framework with the adversarial learning agent will predict these hard-to-discover faults.

## Discussion

The neuroevolutionary training of a population of adversarial agents was successful, as seen by increasing rewards for the agents throughout the training process. In the clustering step of training, diversity was achieved, and the system didn’t converge to a single solution. The top agent’s reward increases steadily at the beginning of training to a maximum reward, but then reaches a plateau. The inconsistent growth from epoch to epoch is a result of fitness sharing in the system due to the random noise added to the parameters on each generation. This is a necessary concession made in the system to not optimize toward a single local minimum.

This approach to creating adversarial agents was successful in reaching its goals, but has several technical limitations. Due to the complexity of the FMU simulation and the corresponding operation software, each simulation takes minutes to run and generates substantial amounts of data. This limits the amount of training that can be conducted and how much data can be stored for the deployment framework.

The digital twins were leveraged to allow for the creation of a true adversarial agent. It allows agents to learn how to break the system components repeatedly without lasting impact, physical or monetary. This process is key when developing intelligent systems that allow for limited or no data collection before deployment. The simulation provides for faster-than-real-time operation, pivotal when attempting to accurately predict and observe possible latent faults.

In this work, a framework for active fault detection is proposed, implemented and demonstrated. A mock space habitat was designed, built, instrumented, and operated to enable the demonstration of the fault detection method developed in this work. A suitable FMEA analysis was conducted to identify damaging faults that would cause irreparable damage to the mock space habitat. A design of experiments was executed to exercise the experimental setup in a variety of operating conditions, normal and faulted. A digital twin model, simulated as an FMU, was validated with the mock habitat’s measurement data.

With a large volume of data collected, multiple ReLAAs were trained to recognize normal and faulted behavior. The training for these ReLAAs was completed in several generations using a neuroevolutionary process so that diversity could be achieved. Then, the agents were deployed to discover damaging actions that could damage the mock space habitat. Because there are multiple agents, a variety of damaging actions were found. With these vulnerabilities discovered, a system operator can then use this information to take proper action, thereby mitigating or preventing faults.

## Supplementary information

Reporting Summary

## Data Availability

The simulation data and experimental data are available from the corresponding author upon request.

## Code availability

The design files, simulation models, and software that support this work are available from the corresponding author upon request.

## References

[1] Beard, S. S. & Starzyk, J. Space tourism market study: orbital space travel & destinations with suborbital space travel. 1–72 (Futron Corp., 2002).

[2] Ballard R, Connolly J (1990). US/USSR joint research in space biology and medicine on Cosmos biosatellites. FASEB J..

[3] UBS Investment Bank. Future of Space Tourism: Lifting Off? Or has COVID-19 stunted adoption? 20 July. https://www.ubs.com/global/en/investment-bank/in-focus/2021/space-tourism.html (2021).

[4] Antonsen EL (2022). Estimating medical risk in human spaceflight. npj Microgravity.

[5] Tang, S. et al. Operation-aware ISHM for environmental control and life support in deep space habitants. 10.2514/6.2018-1365 (2018).

[6] Abid A, Khan MT, Iqbal J (2021). A review on fault detection and diagnosis techniques: basics and beyond. Artif. Intell. Rev..

[7] Daigle, M. et al. A comprehensive diagnosis methodology for complex hybrid systems. *IEEE Transactions on Systems, Man, and Cybernetics – Part A: Systems and Humans* (2010).

[8] Jiang J, Yu X (2012). Fault-tolerant control systems: a comparative study between active and passive approaches. Ann. Rev. Control.

[9] Mustapha S, Lu Y, Ng C-T, Malinowski P (2021). Sensor networks for structures health monitoring: placement, implementations, and challenges–a review. Vibration.

[10] Colombano, S. et al. A system for fault management for NASA’s deep space habitat. *International Conference on Environmental Systems (ICES)* (2013).

[11] Iverson, D. et al. General purpose data-driven system monitoring for space operations. *J. Aerosp. Comput. Inf. Commun.* **9**, 26–44 (2012).

[12] Spirkovska, L. et al. Anomaly detection for next-generation space launch ground operations. *Proceedings of the AIAA SpaceOps 2010 Conference* (AIAA, Huntsville, AL, 2010).

[13] Wang J, Li Y, Gao R, Zhang F (2022). Hybrid physics-based and data-driven models for smart manufacturing: modelling, simulation, and explainability. J. Manuf. Syst..

[14] Rackauckas, C. et al. Universal Differential Equations for Scientific Machine Learning. *Proceedings of the National Academy of Sciences of the United States of America*. 10.21203/rs.3.rs-55125/v1 (2020).

[15] Uhlemann TH-J, Schock C, Lehmann C, Freiberger S, Steinhilper R (2017). The digital twin: demonstrating the potential of real time data acquisition in production systems. Procedia Manuf..

[16] Stanley, K., Clune, J., Lehman, J. & Miikkulainen, R. Designing neural networks through neuroevolution. *Nat. Mach. Intell.* **1**, 24–35 (2019).

[17] Papavasileiou E, Cornelis J, Jansen B (2021). A systematic literature review of the successors of “neuroevolution of augmenting topologies”. Evol. Comput..

[18] Sutton, R. S. & Barto, A. G. *Reinforcement Learning: An Introduction* (MIT Press, 2018).

[19] Such, F. P. et al. Deep neuroevolution: genetic algorithms are a competitive alternative for training deep neural networks for reinforcement learning. Preprint at *arXiv:1712.06567 [cs]* (2018).

[20] Ibrahim, M. Y., Sridhar, R., Geetha, T. V. & Deepika, S. S. Advances in neuroevolution through augmenting topologies – a case study. *2019 11th International Conference on Advanced Computing (ICoAC)* 111–116. 10.1109/ICoAC48765.2019.246825 (2019).

[21] Miller, B. L. & Shaw, M. J. Genetic algorithms with dynamic niche sharing for multimodal function optimization. *Proceedings of IEEE International Conference on Evolutionary Computation* (IEEE, 1996).

[22] Chang P-C, Huang W-H, Ting C-J (2010). Dynamic diversity control in genetic algorithm for mining unsearched solution space in TSP problems. Expert Syst. Appl..

[23] Simon, M. Whitmire, A., Otto, C. & Neubek, D. Factors impacting habitable volume requirements: results from the 2011 Habitable Volume Workshop. *National Aeronautics and Space Administration (NASA), Center for Advanced Space Studies-Universities Space Research Association* (2011).

[24] Caldwell, S. & Dunbar, B. National Aeronautics and Space Administration (NASA). “7.0 Thermal Control.” Updated 4 November 2021. https://www.nasa.gov/smallsat-institute/sst-soa/thermal-control (2021).

[25] Indranil, R., Hafiychuk, V., & Goebel, K. Model-based diagnosis and prognosis of a water recycling system. *IEEE Aerospace Conference* (2013).

[26] Gelernter, D. *Mirror Worlds or the Day Software Puts the Universe in a Shoebox: How Will It Happen and What It Will Mean*. ISBN: 0195068122 (Oxford University Press, Inc., 1991).

[27] International Business Machine Corporation (IBM). How does a digital twin work? https://www.ibm.com/topics/what-is-a-digital-twin.

[28] Functional Mock-up Interface (FMI). https://fmi-standard.org/ (2022).

[29] Kullback S, Leibler RA (1951). On information and sufficiency. Ann. Math. Statist..

