# Supplementary material for: Reinforcement learned adversarial agent (ReLAA) for active fault detection and prediction in space habitats
Source: NPJ Microgravity. 2023 Feb 13;9:15. doi: 10.1038/s41526-023-00252-9 (PMC9925772; doi:10.1038/s41526-023-00252-9)
Supplement: Supplementary file 1 — Reporting Summary [file 41526_2023_252_MOESM1_ESM.pdf]

nature portfolio

Corresponding author(s): Matthew Overlin

Last updated by author(s): Oct 17, 2022

Reporting Summary

Nature Portfolio wishes to improve the reproducibility of the work that we publish. This form provides structure for consistency and transparency in reporting. For further information on Nature Portfolio policies, see our Editorial Policies and the Editorial Policy Checklist.

Statistics

For all statistical analyses, confirm that the following items are present in the figure legend, table legend, main text, or Methods section.

n/a	Confirmed	
☒	☐	The exact sample size (*n*) for each experimental group/condition, given as a discrete number and unit of measurement
☐	☒	A statement on whether measurements were taken from distinct samples or whether the same sample was measured repeatedly
☐	☒	The statistical test(s) used AND whether they are one- or two-sided<br>*Only common tests should be described solely by name; describe more complex techniques in the Methods section.*
☐	☒	A description of all covariates tested
☐	☒	A description of any assumptions or corrections, such as tests of normality and adjustment for multiple comparisons
☐	☒	A full description of the statistical parameters including central tendency (e.g. means) or other basic estimates (e.g. regression coefficient) AND variation (e.g. standard deviation) or associated estimates of uncertainty (e.g. confidence intervals)
☒	☐	For null hypothesis testing, the test statistic (e.g. *F*, *t*, *r*) with confidence intervals, effect sizes, degrees of freedom and *P* value noted<br>*Give P values as exact values whenever suitable.*
☒	☐	For Bayesian analysis, information on the choice of priors and Markov chain Monte Carlo settings
☐	☒	For hierarchical and complex designs, identification of the appropriate level for tests and full reporting of outcomes
☒	☐	Estimates of effect sizes (e.g. Cohen's *d*, Pearson's *r*), indicating how they were calculated

Our web collection on statistics for biologists contains articles on many of the points above.

Software and code

Policy information about availability of computer code

Data collection	The experimental set-up integrates the data collection and operation activities of the physical demonstration system to a sensor and computational hub that can remotely monitor and actuate the system. This computational hub uses NASA's core Flight System (cFS) middleware software architecture to ingest sensor data, process raw voltages to physical values, and record historical data for use by a digital twin. Further developments were also accomplished to allow a terminal user to have remote access to the system, control programmable actuators on the system, and synchronize data to a cloud storage service. The sensor and control hub computer is a Raspberry Pi (RPi) 4b single board computer that communicates with two Arduino Mega microcontroller boards via USB. One microcontroller is used for reading sensor values, and the other is used for issuing commands to the demonstration system's programmable actuators.
Data analysis	Much of the experimental data analysis was completed in python 3.8. Much of the simulated data analysis was completed in Matlab 2020b and python 3.8.

For manuscripts utilizing custom algorithms or software that are central to the research but not yet described in published literature, software must be made available to editors and reviewers. We strongly encourage code deposition in a community repository (e.g. GitHub). See the Nature Portfolio guidelines for submitting code & software for further information.

Data

Policy information about availability of data

All manuscripts must include a data availability statement. This statement should provide the following information, where applicable:

- Accession codes, unique identifiers, or web links for publicly available datasets
- A description of any restrictions on data availability
- For clinical datasets or third party data, please ensure that the statement adheres to our policy

The simulation data and experimental data are available from the corresponding author upon request.

Human research participants

Policy information about studies involving human research participants and Sex and Gender in Research.

Reporting on sex and gender	Not applicable. Human research participants did not participate in this work.
Population characteristics	Not applicable. Human research participants did not participate in this work.
Recruitment	Not applicable. Human research participants did not participate in this work.
Ethics oversight	Not applicable. Human research participants did not participate in this work. (NASA sponsored this work).

Note that full information on the approval of the study protocol must also be provided in the manuscript.

Field-specific reporting

Please select the one below that is the best fit for your research. If you are not sure, read the appropriate sections before making your selection.

☐ Life sciences ☐ Behavioural & social sciences ☒ Ecological, evolutionary & environmental sciences

For a reference copy of the document with all sections, see nature.com/documents/nr-reporting-summary-flat.pdf

Ecological, evolutionary & environmental sciences study design

All studies must disclose on these points even when the disclosure is negative.

Study description	A scaled-down mock space habitat was built as a hardware prototype. The stable, reliable operation of such a habitat is partly achieved with an ability to recognize and predict faults. For these two purposes, a reinforcement learning adversarial agent (ReLAA) is utilized in this work. A ReLAA is trained with experimental data to actively recognize and predict faults. These capabilities are achieved by proposing actions which activate known faults in a system. Instead of issuing these harmful actions to the actual hardware, a digital twin of the mock space habitat is simulated to discover vulnerabilities which would lead to faulted operation. The methods developed in this work will allow for the discovery of damaging latent behavior, and the reduction of false positive and negative fault identification.
Research sample	No living organisms were used in this project whatsoever. In this project, a spare office room was re-purposed to be a mock space habitat. The thermal control system, grey water filtering system, and electrical system were all thoroughly instrumented with sensors. The data collected from this experimental set-up is described as time series data.
Sampling strategy	There were no living organisms in this study whatsoever. The sampled time series data was collected from a variety of sensors in the mock space habitat via an Arduino Mega controller. Section 2.2 in the article may give some relevant information here.
Data collection	(Section 2.2 in the article describes the data collection procedure.)<br>The experimental set-up integrates the data collection and operation activities of the physical demonstration system to a sensor and computational hub that can remotely monitor and actuate the system. This computational hub uses NASA's core Flight System (cFS) middleware software architecture to ingest sensor data, process raw voltages to physical values, and record historical data for use by a digital twin. Further developments were also accomplished to allow a terminal user to have remote access to the system, control programmable actuators on the system, and synchronize data to a cloud storage service. The sensor and control hub computer is a Raspberry Pi (RPi) 4b single board computer that commu-nicates with two Arduino Mega microcontroller boards via USB. One microcontroller is used for reading sensor values,

	and the other is used for issuing commands to the demonstration system's programmable actuators.
Timing and spatial scale	Each data collection started and stopped on the same day. The spatial scale is not relevant. As for the timing scale, the experiments used ranged in duration: 5 minutes - 8 hours. The data was collected/sampled at 10-sec. time steps. On some days, experiments were performed and data was collected. On other days, engineers needed to attend to other duties on the project or other duties on other unrelated projects at PacMar Technologies (FKA Martin Defense Group). Experiments were performed on the following days in 2022:<br>Jan. 13, 14<br>Feb. 2, 11, 23, 24, 25<br>Mar. 8, 9, 10, 11, 14, 15, 17, 18
Data exclusions	In some cases, a sensor would report erroneous information or not be configured correctly. Some sensors needed to be properly configured or replaced. In these cases, the experiment would be re-performed and noted on one of the days in Jan. - Mar. 2022 previously noted.
Reproducibility	In its final state, the mock space habitat operated repeatedly. To ensure reproducibility, engineers working on the project thoroughly documented the hardware design with schematics and a bill of materials. For the software and middleware used in this project, all necessary files were saved and packaged together, so that one may use them. A Software Technology Volume was written to inform a curious scientist or engineer about how to use the software and models.
Randomization	No living organisms were used in this work whatsoever. All sensors were sampled to retrieve time series data. Each sensor's data was not categorized into groups.
Blinding	Blinding was not relevant in this study. No living organisms were used in this study whatsoever. All data was collected from a mock space habitat. Neither the mock space habitat nor any of its sub-systems possess consciousness or any cognitive ability in any form or fashion.

Did the study involve field work? ☐ Yes ☒ No

Reporting for specific materials, systems and methods

We require information from authors about some types of materials, experimental systems and methods used in many studies. Here, indicate whether each material, system or method listed is relevant to your study. If you are not sure if a list item applies to your research, read the appropriate section before selecting a response.

Materials & experimental systems

n/a	Involved in the study
☒	☐ Antibodies
☒	☐ Eukaryotic cell lines
☒	☐ Palaeontology and archaeology
☒	☐ Animals and other organisms
☒	☐ Clinical data
☒	☐ Dual use research of concern

Methods

n/a	Involved in the study
☒	☐ ChIP-seq
☒	☐ Flow cytometry
☒	☐ MRI-based neuroimaging
